# Multiple particle tracking analysis in isolated nuclei reveals the mechanical phenotype of leukemia cells

**DOI:** 10.1038/s41598-020-63682-5

**Published:** 2020-04-21

**Authors:** Diego Herráez-Aguilar, Elena Madrazo, Horacio López-Menéndez, Manuel Ramírez, Francisco Monroy, Javier Redondo-Muñoz

**Affiliations:** 1 Department of Physical Chemistry, Complutense University, 28040 Madrid, Spain; 2 Faculty of Experimental Sciences, Francisco de Vitoria University (UFV), 28223 Pozuelo de Alarcón, Madrid Spain; 3 Department of Immunology, Hospital 12 de Octubre Health Research Institute (imas12), School of Medicine, Complutense University, 28040 Madrid, Spain; 4 Translational Biophysics, Hospital Doce de Octubre Health Research Institute (imas12), 28041 Madrid, Spain; 5 Oncolohematology. Hospital Universitario Niño Jesús, Madrid, Spain; 6 Health Research Institute La Princesa, Madrid, Spain; 7 Lydia Becker Institute of Immunology and Inflammation, Manchester Collaborative Centre for Inflammation Research, University of Manchester, Manchester, M13 9PL UK

**Keywords:** Biophysics, Nuclear organization, Chemical biology

## Abstract

The nucleus is fundamentally composed by lamina and nuclear membranes that enclose the chromatin, nucleoskeletal components and suspending nucleoplasm. The functional connections of this network integrate external stimuli into cell signals, including physical forces to mechanical responses of the nucleus. Canonically, the morphological characteristics of the nucleus, as shape and size, have served for pathologists to stratify and diagnose cancer patients; however, novel biophysical techniques must exploit physical parameters to improve cancer diagnosis. By using multiple particle tracking (MPT) technique on chromatin granules, we designed a SURF (Speeded Up Robust Features)-based algorithm to study the mechanical properties of isolated nuclei and in living cells. We have determined the apparent shear stiffness, viscosity and optical density of the nucleus, and how the chromatin structure influences on these biophysical values. Moreover, we used our MPT-SURF analysis to study the apparent mechanical properties of isolated nuclei from patients of acute lymphoblastic leukemia. We found that leukemia cells exhibited mechanical differences compared to normal lymphocytes. Interestingly, isolated nuclei from high-risk leukemia cells showed increased viscosity than their counterparts from normal lymphocytes, whilst nuclei from relapsed-patient's cells presented higher density than those from normal lymphocytes or standard- and high-risk leukemia cells. Taken together, here we presented how MPT-SURF analysis of nuclear chromatin granules defines nuclear mechanical phenotypic features, which might be clinically relevant.

## Introduction

The nucleus is a central cellular organelle that must alter its physical properties during cellular functions, including gene expression, cell migration, and development in homeostasis and human diseases^1^. The nucleus is composed by the nuclear envelope, nucleoskeletal components, and the nucleoplasm, which contains the DNA and its associated molecules forming the chromatin^2^. The nuclear envelope is mainly composed by nuclear membranes, A- (lamin A and C) and B- (lamin B) lamin types, and other structural proteins that connect the nucleus with the cytoskeleton as LINC complexes^3^. Lamin A/C levels and its ratio to lamin B levels control nuclear deformability and stiffness^4,5^. It has been reported that other nuclear components, as LINC and F-actin binding proteins, control nuclear shape and rigidity^6^. In general, these nuclear changes correlate with more invasive phenotype of tumor cells and higher genomic instability upon cell migration^7,8^.

Chromatin organization is modulated by epigenetic changes that promote chromatin compaction and decondensation according to electrostatic interactions and configurational entropy^9–11^. Several biophysical techniques support that the chromatin conformation alterations contributes to the morphology and the biophysical behavior of the nucleus^12–16^ Abnormalities in nuclear shape and organization occur in a wide range of human pathologies, including cancer^17,18^. Likewise, nuclear morphology has been still used for diagnoses in many biopsies by pathologists^19,20^. Whereas several studies have improved nuclear morphometric experiments to stratify cancer cells^21^, the functional links between the biophysical properties of nuclei from cancer cells and their value in clinics remain elusive.

In the case of acute lymphoblastic leukemia (ALL), novel strategies for prevention and early detection have pointed the critical role of genetic changes leading to nuclear modifications on the molecular pathogenicity of the neoplastic cells^22^. ALL is the most common pediatric malignancy and the leading cause of death in children with cancer^23^. However, it is not known how the nuclei of ALL cells differ from normal peripheral blood lymphocytes (PBL).

In this study, we used a multiple particle tracking (MPT) analysis of chromatin granules to determine coarse-grained descriptors of nuclear mechanics in isolated nuclei from leukemia cells. MPT technique is broadly recognized as a key technology for quantitative analysis of intracellular mechanics^24,25^. Based on the microrheology concept^26^, we have exploited MPT with time-lapsed microscopy images as a phenotyping method with mechanical markers expanded on the principles of micromechanical cell mapping^27,28^. The robustness of MPT relies on the efficiency of the tracking algorithm to ensure the correspondence of the multiple objects and an adequate frame-of-reference for drift-correction between consecutive slides^24,27,29^. We have developed a novel MPT-method based on the SURF algorithm^30^ which determine *in situ* the apparent rheological properties of the cell nucleus by tracking the mobility of nuclear granules. This paper focusses on the relative variations of the apparent nuclear viscosities between different phenotypes in isolated nuclei although we have resolved also the mechanical descriptors in intact cells. By using primary samples obtained from patients with ALL, we observed that leukemia cells present a different density than normal lymphocytes. Moreover, we were able to identify that isolated nuclei from high-risk ALL cells show higher viscosity than standard-risk or normal lymphocytes. Together, our analysis of biophysical traits of chromatin granules defines the mechanical phenotype of isolated nuclei from leukemia cells that might be relevant to stratify patients.

## Results

### Chromatin mobility by Multiple Particle Tracking enhanced upon Speeded-Up Robust Feature detection (MPT-SURF)

**Figure 1:**
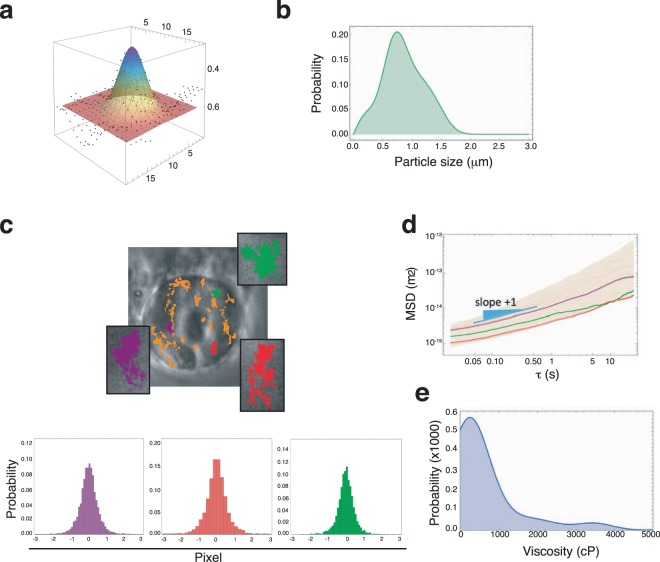
Description of the experimental rationale used for particle tracking microrheology with chromatin grains. **(a**) Spatial profile of a typical chromatin grain with the best fit to a 2D-Gaussian profile. To be eligible for microrheological analysis, a given dynamical trajectory is obligated to conserve apparent grain dimensions. (**b)** Typical distribution of grain sizes in a nucleus, specifically that of Fig. 1**c**. **(c)** Brownian trajectories of selected chromatin grains in a nucleus from Jurkat cell. Three particular trajectories (insets in green, red and purple) were zoomed to show their Brownian nature characterized by a Gaussian distribution of the displacements. (**d)** Variability band of the mean square displacements (MSD trajectories) as calculated from the Brownian trajectories as a function of the lag time ($\tau$). All trajectories were found almost parallel, with a slope unity at short times compatible with free-diffusivity ($MSD \sim \tau$) and an intercept given by the diffusion coefficient ${D}_{eff}$ (see Eq. 1). Variability depended on the different grain sizes (see Fig. 1b), and tthe different environmental microviscosity sensed by every one of those particles. The three highlighted trajectories correspond to the three selected grains in Fig. 1c (equal colors). (**e)** Distribution of the measured values of the apparent viscosities ${\eta }_{app}$ using Eq. (2) with the values of the diffusion coefficient calculated from the best fits with Eq. (1) to the data in Fig. 1d. The apparent particle size $R$ was assumed to equal the measured grain size (Fig. 1a).

Chromatin is packed in nucleosomes folded into 30 nm helical fiber, and this into higher dynamic chromosome territories^31^. Due to its heterogeneity, we considered the possibility to probe coarse-grained chromatin dynamics undergoing confined Brownian motion in a viscoelastic environment^32^. We measured the diffusing trajectories of single granules of chromatin (chromatin “spots”) localized in the equatorial plane of isolated nuclei from Jurkat (a T-ALL cell line) cells (Fig. 1a). To track the positions of the centroids in real time (${r}_{i}(t)$), we designed an adapted SURF (Speeded Up Robust Features) algorithm, which we integrated in a custom-made MPT scheme programmed in Mathematica (Supplementary Notes 1–4 and Supplementary Figs S1 and S2). The MPT-SURF method removed spurious motions due to possible drifts to resolve the collective movement of these particles (Supplementary Fig. S3). We selected for MPT-SURF analysis those granules with diameters between $0.5-1.5\,\mu m$ (Fig. 1b). We also confirmed that the relative size and the optical density of these chromatin granules remained steady during measurements (Supplementary Fig. S4), without any significant change (Supplementary Fig. S5).

The Brownian movement of nuclear granules identified was characterized by a Gaussian profile of displacements (Fig. 1c), which defines diffusing trajectories of mean squared displacements in terms of lag times $\tau$, these are $MSD=\Delta {h}^{2}(\tau )=\sum _{j}{[{r}_{j}(t+\tau )-{r}_{j}(t)]}^{2}$(with the averaging sum extended over all the positions in a time series). We used Jurkat cells (a T-ALL cell line) to isolate the cell nucleus and obtained the Brownian displacements from chromatin spots ($n=72$) (Fig. 1d). Each trajectory was found nearly-free diffusive at enough short-times, where was fitted to the 2D free-diffusion equation^24^:

1 $$\Delta {h}_{\tau \to 0}^{2}=4{D}_{eff}\tau $$

with ${D}_{eff}$ being an effective diffusion coefficient calculated for the corresponding chromatin spot ($MSD\sim {\tau }^{1}$ at $\tau \ll 1s$; Fig. 1d and Supplementary Fig. S6a–c). To test MPT-SURF for performance with *ex-cell* measurements in isolated nuclei, we compared the MPT-SURF analysis in intact Jurkat cells or isolated nuclei (Supplementary Note S4.4). Both types of measurements (ex-cell/in-cell) rendered the Brownian trajectories with the limiting free-diffusion behavior expected at short times (Supplementary Fig. S6b,c); at $\tau \ll 1s$, we observed $MSD\sim {\tau }^{1}$ and $RMSD\sim {\tau }^{1/2}$, as expected. Chromatin diffusivity is hindered by the viscoelastic environment^32^, as we confirmed in our system by the evident confinement of the trajectories (Supplementary Fig. S6b). In addition, we detected hyper-diffusive displacements at long times that correspond to a free-diffusivity breakout ($MSD\sim {\tau }^{\alpha }$ with $\alpha  > 1$ at $\tau  > 1s$; Fig. 1d, Supplementary Fig. S6c). Together, these results indicate that MPT-SURF algorithm enables for detecting Brownian trajectories of chromatin spots with well-defined features expected to remain steady during measurements.

### Apparent chromatin microviscosity as a probe of interphase nuclear mechanics

We determined the distribution of apparent viscosities ${\eta }_{app}$ obtained for each chromatin spot displacement from the Stokes-Einstein relationship^33,34^,

2 $${D}_{eff}=\frac{{k}_{B}T}{6\pi {\eta }_{app}R}$$

where ${k}_{B}$ is the Boltzmann's constant, $t$ the absolute temperature, and $R$ the apparent size of the chromatin spot determined as an optical radius by fitting its intensity profile to a Gaussian function. We defined the apparent viscosity from the tracks of the chromatin spots (including the most probable expectation and the standard deviation; $n\gg 50$, typically) (Fig. 1e). We calculated that the characteristic uncertainty on this phenotypical descriptor (apparent microviscosity) was 30–35% of the averaged value in a single nucleus (summing up experimental error plus data variance due to chromatin heterogeneity). We did not find significant differences between isolated nuclei or these in intact Jurkat cells (Supplementary Fig. S6d). Together, our findings suggest that MPT-SURF analysis of nuclear spots allows to measure the apparent chromatin viscoelasticity in an isolated nucleus.

### Chromatin microrheology reveals the n ucleus mechanics as a Voigt-like body with a regulated viscoelasticity

**Figure 2:**
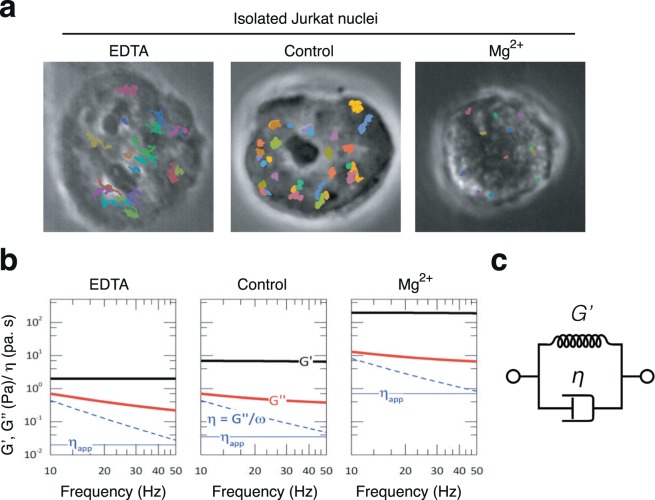
Chromatin viscoelasticity of isolated nuclei from Jurkat cells. (**a**) Representative phase contrast images of Jurkat nuclei analyzed by adding MgCl_2_ or EDTA. (**b)** Experimental values of the viscoelastic moduli of a representative chromatin grain in a Jurkat nucleus as a function of the shearing frequency (inverse Brownian time; $\omega =1/\tau$); $G{\prime} (\omega )$ were the shear rigidity modulus (black lines), and $G{\prime\prime} (\omega )=\omega \eta$ the loss modulus (red lines), which was determined by the frequency-dependence of the effective viscosity $\eta (\omega )$ (dashed blue lines). Straight blue lines show the constant values of apparent viscosity measured from the Brownian diffusivities. The apparent viscosity ${\eta }_{app}$ defined the instantaneous limit of the dynamic viscosity $\eta (\omega )$ at high $\omega$, which represented the short-time limit of free-diffusivity (see Fig. 1d). (**c)** The Voigt-like rheological model described the chromatin as an elastic spring of rigidity $G{\prime}$ coupled in parallel with a damping element of viscosity $\eta$., which represented together a soft viscoelastic body with a mechanical e lowest.

We focused on the frequency dependence of the apparent viscoelastic parameters obtained from an effective fluctuation-dissipation scheme^25,35,36^, which defines the apparently linear mechanic response of the nucleus. Given the complex value of the linear viscoelastic modulus^35^ $\tilde{G}(\omega )=G{\prime} (\omega )+iG{\prime\prime} (\omega )$, with apparent values of the storage modulus $G{\prime} (\omega )$, loss modulus $G{\prime\prime} (\omega )=\omega \eta (\omega )$ and shear viscosity $\eta (\omega )$ expressed as a function of the frequency of the chromatin motions $(\omega )$, the generalized Stokes-Einstein relationship in Eq. (2) can be rewritten as follows^35,37^:

3 $$\tilde{G}(s)=\frac{{k}_{B}T}{\pi Rs\langle {\tilde{r}}^{2}(s)\rangle }$$

where $\tilde{G}(s)$ is the Laplace transform of $\tilde{G}(\omega )$, and ${\tilde{r}}^{2}(s)$ the Laplace transform of the diffusive trajectory $r{(t)}^{2}$, with $s$ being the Laplace frequency (see Methods). The thermal force involved was weak, therefore the passive microrheological response detected by MPT-SURF was guaranteed in the linear region of the strain-stress relationship that underlies Eq. (3). Using this microrheological relationship, we studied the apparent viscoelasticity of isolated nuclei incubated at different conditions (Fig. 2a). Figure 2b shows a representative frequency dependence of the viscoelastic parameters calculated by MPT-SURF; $G{\prime} (\omega )$ was obtained as the real part of the complex modulus $\tilde{G}(\omega )$, $G{\prime\prime} (\omega )$ was the imaginary part, and the shear viscosity $\eta (\omega )=G{\prime\prime} (\omega )/\omega$ was compared with the value of the apparent viscosity ${\eta }_{app}$, as measured from the effective diffusion coefficient (See Eq. (2)). In the Fourier frequency domain probed in the experiments (corresponding to the inverse times of the Brownian trajectories tracked), the apparent value of the storage modulus in isolated nuclei remained essentially constant (typical value $G{\prime} =8\pm 3P$a; $\,N=17)$ (Fig. 2b; central panel). This value was similar to the low rigidity of the cytoplasm^33^ and compatible with the shear rigidity of soft biological gels^38,39^. The apparent value of the loss modulus was constant with a lower value, $G{\prime\prime} \approx G{\prime} /10$, which slightly decreased with increasing frequencies. Due to the rheological behavior observed ($G{\prime} \gg G{\prime\prime} \sim {\omega }^{0}$), we identified the chromatin as a Kelvin-Voigt material constituted by an elastic element (the shear rigidity) coupled in parallel with a viscous damper (the shear viscosity) (Fig. 2c). In this Voigt-like system, viscoelasticity was such that viscous losses were significantly smaller than rigidity ($G{\prime\prime} \ll G{\prime}$), which defined viscous creep as the preferred rheological channel to undergo chromatin deformations. Chromatin motion might depend on the viscous channel undergoing displacements at a velocity limited by the local viscosity. Therefore, we focused on the apparent viscosity ${\eta }_{app}$ as the rheological descriptor with a phenotypical value. We defined that the dynamic value of the shear viscosity decreased with frequency, and reached a limiting value $\eta (\omega )\to {\eta }_{app}$ at high $\omega$, which was compatible with the apparent value determined from the diffusive part of MSD trajectories (Fig. 2b; central panel). Moreover, we confirmed by MPT-SURF that nuclei after isolation or in intact cells showed similar viscosity (Supplementary Fig. S6). We also measured the nuclear area from isolated nuclei and in intact cells (Supplementary Fig. S7). As we expected from previous reports^40^, the isolation process induced nuclear shrinking, although this did not affect the microrheology quantification. Figure 2a shows that nuclei incubated with EDTA swelled in comparison to control (untreated) nuclei, whilst the presence of Mg^2+^ induced nuclear shrinking. Together, our results suggest that MPT-SURF might serve to characterize the mechanical phenotype of isolated nuclei under different conditions.

### Effect of osmotic stress in acute lymphoblastic leukemia cells

**Figure 3:**
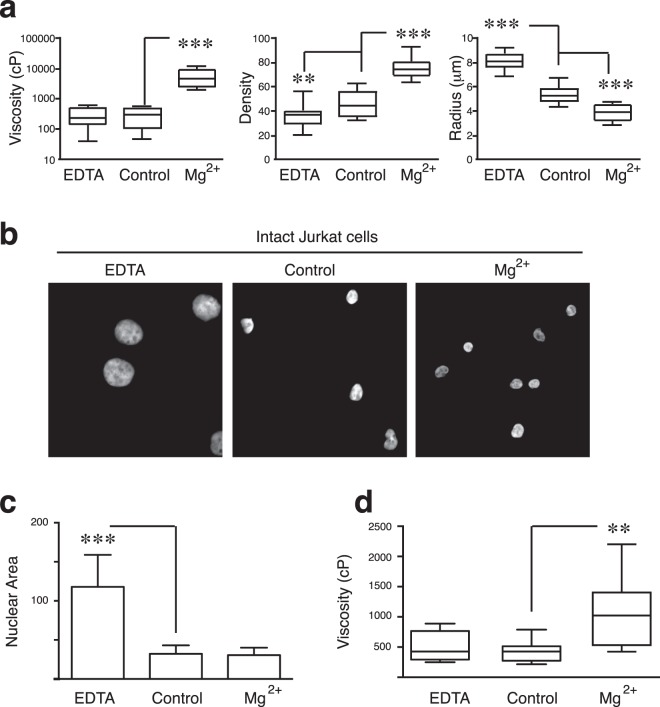
Viscosity alterations induced by osmotic stress of nuclei upon isolation or in intact cells. (**a**) Comparative statistics of the averaged values of the apparent viscosity, optical density and radius measured for a population of Jurkat nuclei at the three different conditions considered. (**b**) Jurkat cells were cultured in the presence of MgCl_2_ (7.5 mM) or EDTA (1 mM) for 24 h. Then, cells were fixed, stained with Hoechst and analyzed by confocal microscopy. (**c**) Graph shows the nuclear areas from (**b**). (**d**) The apparent nuclear viscosity for a population of Jurkat cells cultured as in (**b**) were analyzed by MPT-SURF. **P < 0.01.

As expected from Fig. 2a, the addition of Mg^2+^ to isolated nuclei promoted nuclear shrinking, significantly smaller area, and bigger viscosity and density than control (untreated) nuclei (Fig. 3a). In contrast, EDTA promoted nuclear swelling and reduced the nuclear density compared to untreated conditions; however, no statistically differences of the viscosity were detected (Fig. 3a). To address whether these osmotic effects on intact cells might promote *in situ* nuclear changes detected by MPT-SURF, we cultured Jurkat cells in high (Mg^2+^) or low (EDTA) levels of divalent cations, which have been reported to increase heterochromatin levels in breast cancer cells^41^. Firstly, we demonstrated that Mg^2+^ addition did not alter the nuclear area of Jurkat cells, whilst EDTA treatment significantly increased it (Fig. 3b,c). Using MPT-SURF measurements, we analyzed the viscosity for nuclei of Jurkat cells treated with Mg^2+^ (Supplementary Fig. S8). For isolated nuclei, the diffusing trajectories displayed a very relevant drop after treatment with Mg^2+^, which revealed significantly slower chromatin mobilities upon osmotic compaction (Supplementary Fig. S8). We confirmed that Mg^2+^-treatment of isolated nuclei of Jurkat cells promoted higher apparent nuclear viscosity than control cells, whilst EDTA addition didn’t show any difference (Fig. 3d).

**Figure 4:**
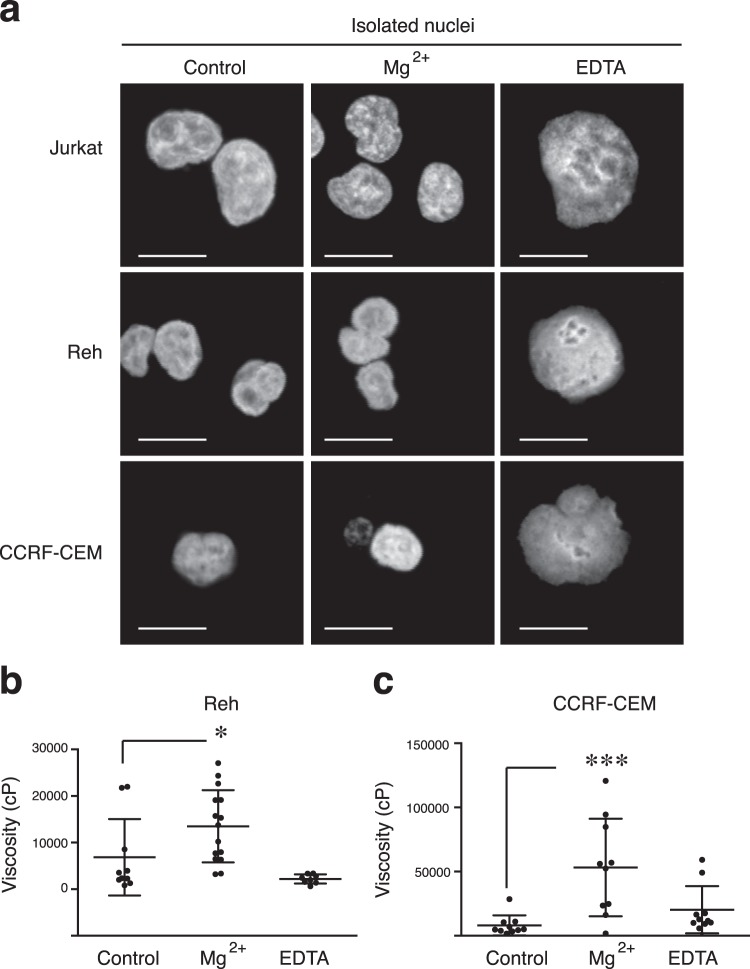
Swelling and shriking conditions promote change in the nuclear shape and viscoelasticity of ALL cell lines. (**a**) Representative images of isolated nuclei from Jurkat and CCRF-CEM (T-ALL) and Reh (B-ALL) cells upon MgCl_2_ or EDTA addition for 5 min. Then, nuclei were fixed, stained with Hoechst and analyzed by confocal microscopy. (**b**,**c**) Comparative statistics of the averaged values of the apparent viscosity measured for a population of Reh (Fig. 3b) or CCRF-CEM (Fig. 3c) nuclei at the three different conditions considered. *P < 0.05.

We expanded our analyses to other B-ALL (Reh) and T-ALL (CCRF-CEM) leukemia cell lines to demonstrate the value of the MPT-SURF analysis of the nuclear microrheology as a quantitative probe of mechanical phenotype. We confirmed that the addition of Mg^2+^ diminished the nuclear morphology, whilst EDTA increased the nuclear shape compared to control conditions of ALL cell lines studied (Fig. 4a). By tracking nuclear chromatin spots from isolated nuclei, we quantified the apparent viscosity of Reh and CCRF-CEM cells. We confirmed that Mg^2+^ addition increased slightly the nuclear viscosity of Reh cells (Fig. 4b) and significantly of CCRF-CEM cells (Fig. 4c). Together, our data demonstrate the general value of MPT-SURF as a probe to detect chromatin compaction/fluidization in the nucleus of a leukemia cell.

### Mechanical phenotype of isolated nuclei of primary leukemic cells

**Figure 5:**
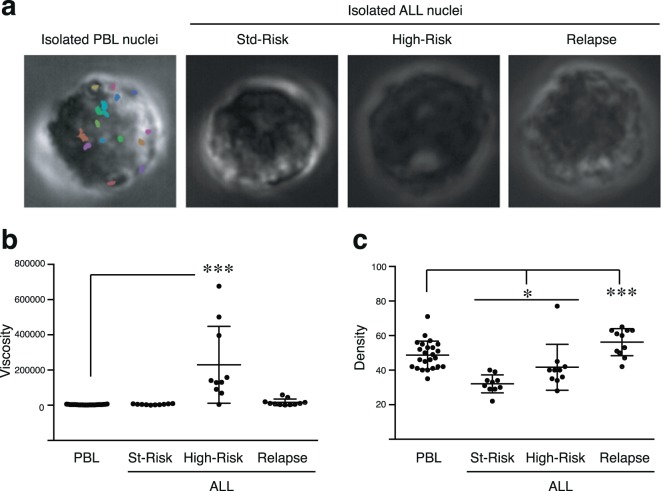
ALL cells present different biomechanical properties according to their clinical feature meassured by passive microrheology. (**a**) Representative phase contrast images of isolated nuclei from normal (PBL) and leukemic ALL cells. ALL cells were stratified according to SHEOP-PETHEMA 2013 in Standard- (St) or High-(High) risk groups and relapsed ALL cells. (**b**) The apparent viscosity from nuclei in Fig. 4a was determined by passive microrheology. (**c**) The optical density from nuclei in Fig. 4a was measured by passive microrheology. *P < 0.05; ***P < 0.001.

ALL patients are commonly stratified according to age, genetic abnormalities, leukocytes in blood count, type of ALL, MRD (minimal residual disease) after induction, etc^42^. To further understand the clinical relevance of MPT-SURF technique, we isolated nuclei from PBL, or cancer ALL cells from patients stratified according to SEHOP-PETHEMA (Spanish Program for the Treatment of Hematologic Diseases) 2013 protocol in standard-or high-risk groups (Fig. 5a). Isolated nuclei of high-risk group of ALL cells presented a significant higher viscosity compared to the other conditions (Fig. 5b). Then, we explored by MPT-SURF the *in situ* nuclear viscosity of intact ALL cells from patients. We observed that cells from a high-risk stratified patient presented a trend to higher nuclear viscosity than standard-risk or relapse ALL cells (Supplementary Fig. S9). Remarkably, isolated nuclei from relapsed ALL cells had higher nuclear density than normal PBL, whilst standard- and high-risk group showed significant lower nuclear density (Fig. 5c). Together, these data indicate that leukemic cells present aberrant mechanical properties in their nuclei that might be related to clinical aggressiveness and/or resistance to chemotherapy.

## Discussion

Chromatin compaction depends on histone packing and intranuclear electrostatic forces^31^. Dynamic changes in chromatin structure control its organization and mobility^43^, and promote auxetic nuclei^44^. Previously, several studies have defined how lamins control the nuclear stiffness and influence on several cell functions (as cell cycle, differentiation, etc.) involved in human pathologies^4–8^. Chromatin viscosity is an emerging actor that contributes to the nuclear mechanics of the cell^45^. Here, we have developed a fast and performant MPT-SURF algorithm that enables for detecting Brownian trajectories of chromatin spots and determining the viscoelastic properties associated to the chromatin configuration of isolated nuclei from normal and leukemia cells.

Nuclear isolation might influence on the chromatin structure and disrupt cytoskeletal bridges between the nucleus and the cell body. Interestingly, nuclear isolation induces a stress-stiffening in the nuclei^40^; however, it has been extensively reported that the nuclear alterations still allow to measure changes in the physical properties of nuclei isolated or in intact cells^45–47^. It has been reported different methods for single nucleus isolation that allows to study the contribution of the chromatin on the mechanical properties of the nucleus as an independent entity^48–51^. Here, we present a MPT-SURF analysis that can be used on fresh biological samples and allow us to determine the mechanical phenotype of isolated nuclei as a parametric setting constituted by the nuclear viscosity, the stiffness and the optical density.

Although the dynamic viscosity of cells has been widely studied^52,53^, the nuclear viscosity of isolated nuclei remains still quite unexplored. Here, we have focused on the local Brownian-like displacements in isolated nuclei of nuclear granules and their apparent diffusion in a soft medium within seconds. These small fluctuations were thermally driven, as deduced from the Gaussian distribution of the displacements and the viscoelastic character of heterogeneous nucleus with dense “spots” undergoing confined Brownian motion. Our results align with the rheological behavior revealed in experiments of protein mobility in chromatin measured by fluorescence correlation spectroscopy (FCS)^32^. Accordingly, previous findings support that the temperature, pH, and salt conditions control the elastic material behavior and volume changes of isolated nuclei^54^. A conceptually similar method has been used with integral nuclei of human HeLa cells using fluorescently labelled histones to track chromatin displacements^55^.

We have confirmed that nuclear shrinking under Mg^2+^ addition causes significant chromatin condensation followed by a high increase of the chromatin viscosity. Conversely, Mg^2+^ depletion (by EDTA) produces visible nuclear swelling. Our results demonstrate the dynamical equivalence between the diffusing behavior of the chromatin granules tracked in both isolated and *in situ* nuclei; although MPT-SURF analysis determined that osmotic stress in living cells promoted smaller mechanical changes than in isolated nuclei. Probably, this is due to regulation mechanisms in the whole cell, through mechanosensitive ion channels able to regulate the nuclear compaction and heterochromatin formation due to changes in the composition of the extracellular medium^41^. Also, we cannot discard the contribution of the cytoskeleton in living cells, which is a major actor in nuclear deformation and strain recovery^56^. Interestingly, we did not observe statistically significant decrease of the nuclear viscosity upon EDTA addition in isolated nuclei nor intact cells, although we observed that some specimens fluidized. This difference in the average viscosity might be due to the heterogeneous distribution of the EDTA-disentangled chromatin (most possibly euchromatin that is not specifically selected in our method as mobile spots), and to the spatial distribution of the chromatin in chromosomal territories and topologically associating domains (TADs)^57^. In general, using intact cells instead of isolated nuclei present the advantage of a more physiological context, which preserves the cytoskeletal and cellular connections; although it makes more difficult to discern the contribution of each component and, in our system, to identify particular properties according the clinical stage of the sample.

It is known that the biomechanical properties of cancer cells can define their phenotype^58,59^. Cancer cells respond to physical forces presented in the tumor environment by controlling the mechanical properties of their nuclei^60^. Interestingly, invasive phenotypes of cancer cells are often softer than normal cells in order to migrate through endothelial barriers and invade other tissues^61^. Recently, it has been reported that the mechanical properties of the nucleus depend on the substrate elasticity and the invasiveness of the cancer cells^62^. In this scenario, new biophysical techniques must be implemented to obtain a quantitative diagnosis independent of the subjective view or interpretation of the pathologists^63^. We found higher nuclear viscosity of high-risk ALL cells than normal PBL. Interestingly, for nuclear density we observed lower values in Standard- and High-Risk ALL cells than in PBL, whilst the relapsed ALL cells presented an increment in the density. A plausible explanation for the differences found is that ALL cells might present an aberrant chromosomal density compared to normal PBL. The implementation of new technologies for diagnoses usually requires further validation by increasing the number of samples, using different subsets of patients and healthy donors. Together, our results present quantitative differences in the viscosity and density of isolated nuclei from leukemia cells with different prognosis. Given the clinical interest for diagnosis, our findings facilitate the possibility to develop new tools for prognosis prediction of cancer cells.

## Methods

### Primary samples and cell lines

The ALL cell lines Jurkat, CCRF-CEM, Reh were obtained from Dr. Ramírez and cultured in RPMI 1640 with L-glutamine and 125 μM Hepes (Sigma Aldrich, St. Louis, MO, USA) with 10% fetal bovine serum (FBS, Sigma-Aldrich). Primary human PBL were isolated from buffy coats of healthy anonymous donors (Blood Bank, Hospital Gregorio Marañón) after depletion of the monocyte fraction with CD14 microbeads. Primary samples from ALL patients under 14 years old were obtained with informed consent for research purposes, and the procedures were approved by the Institutional Review Boards of the Hospital General Universitario Gregorio Marañón (Epicon) and the Hospital Universitario Niño Jesús (R0070/15). ALL diagnosis and treatment were defined according to SEHOP-PETHEMA 2013 (Spanish Program for the Treatment of Hematologic Diseases).

### Immunofluorescence

Nuclei from Jurkat cells were isolated using a hypotonic buffer A (10 mM HEPES, 10 mM KCl, 1.5 mM MgCl_2_, 0.34 M sucrose, 10% (v/v) glycerol, 1 mM DTT and Roche protease inhibitor) and 0.5% of NP-40 followed by vortexing for 15 sec and centrifugation for 5 min at 4°C 3,500 g. Nuclei were resuspended in TKMC buffer (50 mM Tris pH 7.5, 25 mM KCl, 3 mM MgCl_2_, 3 mM CaCl_2_, and proteinase inhibitors) and sedimented onto poly-Lysine coated slides (Thermo Scientific). Nuclei were incubated or not with EDTA, 3 mM (swelling condition) or MgCl_2_ 3 mM (shrinking condition) for 5 min. Then, nuclei were fixed with 4% formaldehyde in PBS (10 min), permeabilized with 0.5% Tx-100 in PBS (5 min) and stained by Hoechst 33342. For intact cells, Jurkat cells were cultured in the presence or not of EDTA (1 mM) or MgCl_2_ (7.5 mM) for 24 h. Then, cells were analyzed by MPT-SURF or fixed, permeabilized and the nucleus stained by Hoechst 33342. Nuclear shape was analyzed by SPE confocal microscopy with an objective ACS-APO 40x NA 1.30 oil immersion. Quantification of nuclear area were determined with Fiji.

### Time-lapse Video Microscopy (TLVM)

Intact cells or isolated nuclei from cells cultured in suspension were washed in isotonic conditions and diluted in TKM buffer. Then, isolated nuclei were deposited onto poly-Lysine coated glass slides and imaged in a phase contrast inverted microscope (NikonEclipse2000Ti) equipped with a 100 W TI-12 DH Pillar Illuminator, an LWD 0.52 collimator, and a 100× oil immersion objective (PlanApoVC, N.A. 1.4; Nikon). Tracking movies of nuclear particles from at least 6 isolated nuclei or cells were captured with a FASTCAM SA3 camera (Photron), with an effective pixel size of 50 × 50 nm^2^. To provide optimal signal-to-noise ratio (SNR), the movies were recorded during 10 s of tracking time at a sampling frequency of 512 Hz (5120 frames).

### Multiple particle tracking of nuclear particles using Speed-Up Robust Feature detection (MPT-SURF)

Time-resolved images from nuclei were analyzed with an MPT-SURF code generated with Mathematica software (Wolfram Research) available as Supplementary Information (Supplementary Notes 1–3). Dense nuclear grains were identified as highly-contrasted objects with a symmetric 2D-Gaussian intensity profile of intensity significantly larger than the averaged background (see Supplementary Note 1). The instantaneous position of every nuclear particle was identified as the position of the maximum of the fitted Gaussian profile; for a particle $i$ placed in a Cartesian frame of reference, we recorded as a function of time $t$: (*i)* two-dimensional coordinate ${r}_{i}(t)=({x}_{i},{y}_{i})$ (corresponding to center of the Gaussian profile), (*ii)* a circular-like diameter ${D}_{i}(t)$ (corresponding to the Gaussian full width at the half maximum), and (*iii)* the intensity ${I}_{i}(t)$ (as the integrated area of the 2D-Gaussian profile). Spot-tracer displacements between two consecutive frames were evaluated by using the SURF feature detection algorithm^30^ (see Supplementary Notes 2, 3 for further description). The instantaneous centroid of these spots was evaluated as the position of the center-of-mass at a given time $t$, this is ${r}_{0}(t)=\sum _{i}{r}_{i}(t){I}_{i}(t)/\sum _{i}{I}_{i}(t)$, using the optical density ${I}_{i}$ as a weighting factor. Troubleshooting was performed by discarding spots with consecutive coordinates varying larger than a 50% of the previous displacement, and more than 10% in the apparent size characteristics (both diameter ${D}_{i}$ and intensity ${I}_{i}$). Larger variations in the apparent size were interpreted as either spurious spot exchanges, or off-plane defocusing giving rise to actual 3D-contributions to the particle displacements. Finally, a coordinate drift correction was performed to the whole set of coordinates at every frame by applying a geometrical rigid transform, *via* singular-value decomposition, which maximized the alignment of the tracers between two consecutive frames and preserves both size and shape. The 2D-acceptable Brownian trajectories drift-corrected by the motion of the center of mass ${r}_{i}^{{\prime} }(t)={r}_{i}(t)-{r}_{0}(t)$ where then processed to get the trajectory of mean square displacements as a function of the lag time $\tau$; for the particle $i$, these is $MS{D}_{i}(\tau )=\sum _{j}{[{r}_{i}^{{\prime} }({t}_{j}+\tau )-{r}_{i}^{{\prime} }({t}_{j})]}^{2}/n$, where the sum was calculated along a given time series ${t}_{j}=j\delta t$, with $j$ = 1, 2…$n$ describing the discrete steps of timelength $\delta t$. Then, by exploiting the 2D-diffusion equation $MS{D}_{i}(\tau )=4{D}_{eff}\tau$, the diffusion coefficient corresponding to every trajectory was computed as the slope ${D}_{eff}$ of the linear fit. Further, the apparent viscosity ${\eta }_{app}$ was estimated using the Stokes-Einstein relationship for sticking conditions, ${D}_{eff}={k}_{B}T/6\pi {\eta }_{app}R$, where ${k}_{B}T$ is the thermal energy, and $R=D/2$ the apparent size of the nuclear particle. The average value calculated in a given specimen over a collection of acceptable nuclear particles (normally higher than 10), was the quantity assumed with phenotyping value (average value in Fig. 1e).

### Laplace-transform microrheology

The Laplace-transform must be performed to evaluate the viscoelastic modulus from the generalized fluctuation-dissipation relationship in Eq. (3). However, rather than a direct evaluation with a high computational cost and a high error from numerical approximations, we accounted both for the shear modulus moduli and the phase angle in polar notation $\tilde{G}(i\omega )={G}_{d}(\omega )\,exp[i{\rm{\delta }}({\rm{\omega }})]$ through the approximate analytic relation^70^; for the modulus, we get:

$${G}_{d}({\rm{\omega }})\approx \frac{2{k}_{B}T}{3\pi R\langle \Delta {R}^{2}({\rm{\tau }})\rangle \,\Gamma \left(1+\frac{d\,ln\,\langle \Delta {R}^{2}(\tau )\rangle \,}{d\,ln\,\tau }\right)}$$

where ${\rm{\tau }}=\,1/{\rm{\omega }}$ and $\Gamma$ being the gamma function; and for the phase:

$$\delta (\omega )\approx \frac{\pi }{2}\left(\frac{d\,ln{G}_{d}(\omega )}{dln(\omega )}\right)$$

### Statistical analysis

Student t test (two tailed Mann-Whitney non-parametric test) or ANOVA (two tailed Kruskal-Wallis non-parametric test) were used for between-group analysis. For all analyses, statistical calculations were performed using Prism 6.0 Software (GraphPad Software, Inc. La Jolla, CA, USA), and p-values <0.05 were considered statistically significant.

### Software availability

The software generated for the microrheological analysis is available from Dr. D. Herráez-Aguilar and F. Monroy upon request.

Written informed consent was obtained from the parents or legal guardians of all participants and from the participants themselves if aged 12 or more years.

## Supplementary information

Supplementary Information.
