# Supplementary material for: Multiple particle tracking analysis in isolated nuclei reveals the mechanical phenotype of leukemia cells
Source: Sci Rep. 2020 Apr 21;10:6707. doi: 10.1038/s41598-020-63682-5 (PMC7174401; doi:10.1038/s41598-020-63682-5)
Supplement: Supplementary file 1 — Supplementary Information. [file 41598_2020_63682_MOESM1_ESM.docx]

**Supplementary Information**

**Multiple particle tracking analysis in isolated nuclei reveals the mechanical phenotype of leukemia cells.**

D.Herráez-Aguilar^1,2^, E. Madrazo^3^, H. López-Menéndez^1^, M. Ramírez^4,5^, F. Monroy^1,6,*^ and J. Redondo-Muñoz^3,7,*^

^1^Department of Physical Chemistry, Complutense University, 28040 Madrid, Spain. ^2^ Faculty of Experimental Sciences, Francisco de Vitoria University (UFV), 28223 Pozuelo de Alarcón, Madrid, Spain. ^3^Department of Immunology, Hospital 12 de Octubre Health Research Institute (imas12), School of Medicine, Complutense University, 28040 Madrid, Spain. ^4^Oncolohematology. Hospital Universitario Niño Jesús, Madrid, Spain. ^5^Health Research Institute La Princesa, Madrid, Spain. ^6^ Translational Biophysics, Hospital Doce de Octubre Health Research Institute (imas12), 28041 Madrid, Spain. ^7^Lydia Becker Institute of Immunology and Inflammation, Manchester Collaborative Centre for Inflammation Research, University of Manchester, Manchester M13 9PL, UK.

*Corresponding authors: [monroy@ucm.es](mailto:Monroy@ucm.es); [javredon@ucm.es](mailto:javredon@ucm.es)

**Supplementary Note 1.**

**Supplementary Note 2.**

**Supplementary Note 3.**

**Supplementary Note 4.**

**Supplementary Note 5.**

**Supplementary Figure S1.**

**Supplementary Figure S2.**

**Supplementary Figure S3.**

**Supplementary Figure S4.**

**Supplementary Figure S5.**

**Supplementary Figure S6.**

**Supplementary Figure S7.**

**Supplementary Figure S8.**

**Supplementary Figure S9.**

**References**

**Supplementary Note 1. Particle tracking for measuring apparent chromatin viscoelasticity: A possible description of the nuclear mechanical phenotype.** We have studied the apparent mechanical properties derived from the Brownian motion of quasi-spherical granules embedded in the viscoelastic chromatin, those we called “chromatin spots” as they presented a quasi-spherical shape and a constant size required for a particle tracer during measurements [^[[1]](#endnote-1)^]. The general method of intracellular multiple particle tracking (MPT) with endogenous granules was introduced by Wirtz and cols. [1,^[[2]](#endnote-2)^]; however, it had been previously proposed to study the mechanical structure of the cytoplasm [^[[3]](#endnote-3)^] and the cytoskeleton [^[[4]](#endnote-4)^]. In their seminal paper, Wirtz and cols. exploited lipid droplets suspended in the cytoplasm as tracer particles for mapping mechanics in living cells. This MPT method has been also sucessfully exploited to study the motions of the small granules percolated in the viscoelastic media, as the cytoskeletal network [^[[5]](#endnote-5)^] and concentrated DNA gels [^[[6]](#endnote-6)^]. In parameteric terms, the microviscosity and the viscoelastic spectrum can be calculated through an effective Stokes-Einstein relationship and the Fourier-transform of the Brownian displacements, respectively [1,5]. The Wirtz’s method is currently a standard to extract mechanical information by exploiting the heterogeneity of a system from MPT data [^[[7]](#endnote-7)^], being considered a well-known method to study intracellular dynamics (see Refs. [^[[8]](#endnote-8)^-,^[[9]](#endnote-9)^,^[[10]](#endnote-10)^] for extensive reviews).

Here, we measured the apparent viscoelasticity of the nucleus as derived from the Brownian motion of individual quasi-spherical spots of chromatin embedded in a viscoelastic material. These loosely-percolated particles were selected to appear during measurements with a constant spherical shape and size and moving distances comparable to their radii. Spots tracking served to determine the local mobility of chromatin, and thus to quantify an apparent value of “microviscosity” by the Stokes-Einstein relationship. Accordingly to spots mobility, the nucleus might show a lower or higher viscosity according to the elevated or reduced mobility of chromatin spots, respectively. Spreading of chromatin spots in the nucleus might indicate chromatin stiffening characterized by a finite shear rigidity thus behaving as a soft solid. To describe the mechanical phenotype of each single nucleus, the average values of the apparent viscoelastic parameters were calculated over the ensemble of chromatin spots selected for multiple particle tracking under the adequate processing that we are describing below (Supplementary Figure S1a).


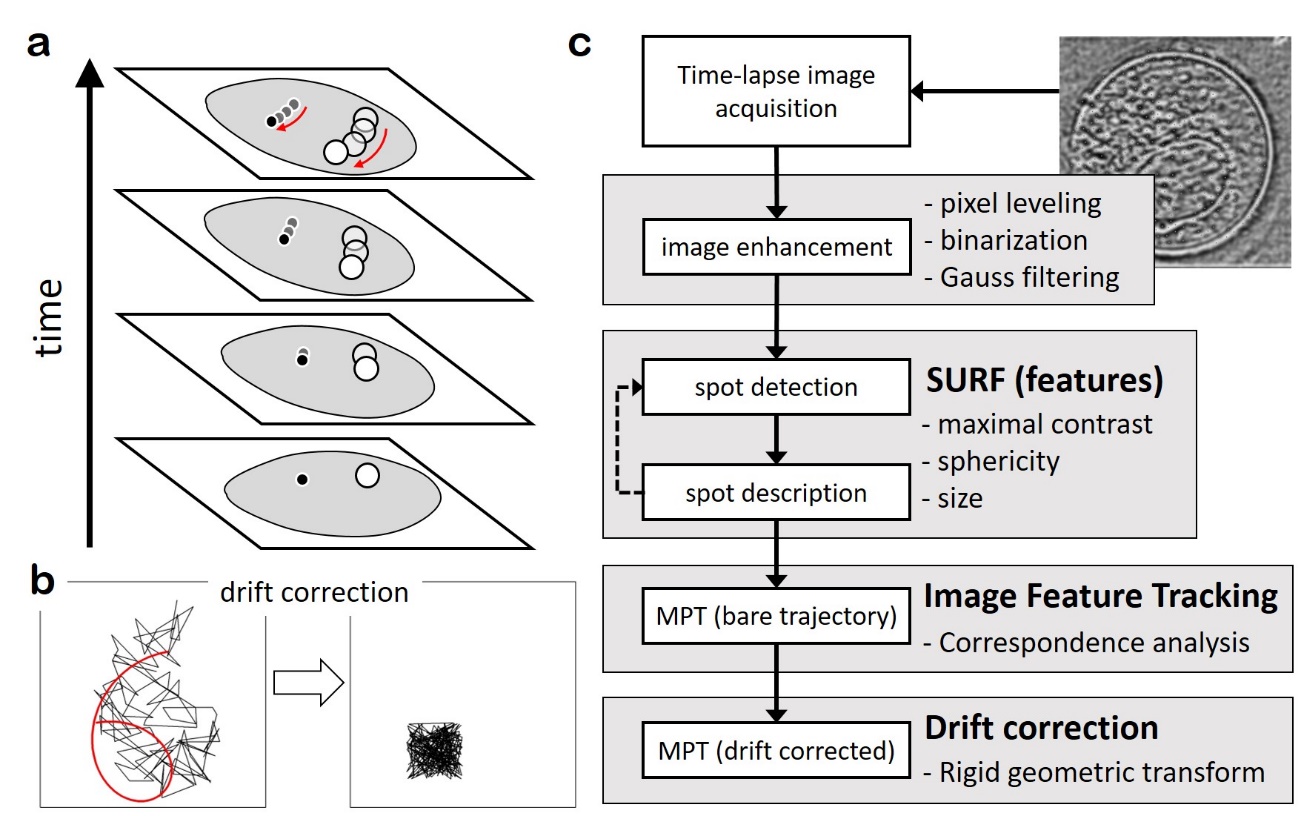


**Figure S1. (a)** Concept for time-lapse microscopy as a probe of motion of featured key-points (or spots) in a living cell. For adequate MPT with chromatin spots in cellular nuclei, multiple spots have to be tracked with invariant features during measurements. The total displacements of these spots results from the combination of their intrinsic motions plus the extrinsic translations and rotations of the nucleus as a rigid object (drift). **(b)** Cartoon on the drift correction of an apparently Brownian trajectory drifted by a translation-rotation drift marked as a straight red line (left panel; before correction). Only the stochastic component remains after drift-subtraction (right panel; after correction). **(c)** Flow-diagram of the MPT-SURF computational schema developed here for the digital processing of image stacks of cellular nuclei recorded from time-lapse microscopy (see Supplementary Note 2 for details).

**Supplementary Note 2. Multiple particle tracking (MPT) with speed-up robust features (SURF).** A robust MPT schema required a computer vision-based correspondence analysis to ensure the unequivocal description of the traced particles. This analysis required also the detection of every single particle trajectory at a level of performance high enough to discard spurious drifts and possible inter-particle exchanges (Supplementary Figure S1b) [^[[11]](#endnote-11)^,^[[12]](#endnote-12)^]. To optimize this MPT analysis on a routine application of computational image processing, we developed our own software for MPT using a pre-validated algorithm of particle detection and image correspondence adapted to the required performance. We used a broadly-used algorithm for real-object recognition at Speeded Up Robust Features (SURF) [^[[13]](#endnote-13)^], which represents a good option for point dectection in terms of repeatability, distinctiveness and robustness [16]. The SURF algorithm is available as a routine that supported easy-integration and assembly in a custom-made MPT scheme programed in MATHEMATICA (Wolfram Research). In our MPT-SURF implementation, we followed a similar standard to that determined by Jaqaman and Danuser [^[[14]](#endnote-14)^], which consists of four modules of computational analysis (Supplementary Figure S1c and Supplementary Note 3).

**Supplementary Note 3. Description of the MPT-SURF algorithm used in this work.**

***Module 1:* Contrast enhancement.**For each frame in a 1-minute sequence (typically 120,000 frames recorded at 2,000 fps; 1024 pixel x 1024 pixel resolution), we performed the following processing steps:

- 1. *Pixel leveling:* Rescaling of the intensity levels in the image by rescaling them to cover the range from 0 to 1. In this step, we performed a local correction of the intensity histogram by using the MATHEMATICA function ImageAdjust[*image*], which adjusts pixel values in the visible range 0-1 by shifting possible outscaled values.
  2. *Pixel binarization:* To better segmentate the contrasted spots present in the image foreground, each pixel was binarized to 0/1 (black/white) by following a local adaptative scheme that accounts for the intensities of its neighborhood as defined by a square box around the pixel (typically, we consider $11\times11$ or $21\times21$ boxes). We used the function LocalAdapatativeBinarize[*image, r*], which determines the binarization threshold in a local box of “radius” *r* where the function calculates the local mean of the intensities over the neighborhood boxes centered on each pixel (typically, *r* = 5 or 10). By defect, the function assigns 0 to mean values below 0.5 and 1 above this threshold.
  3. *Gauss filtering:* Finally, to render derivable the images, we applied a Gaussian filter that reduces noise and smooths the previously binarized image, this is GaussianFilter[*image, r*]; we used the same definition for the neighborhood box as above, *r* = 5 (or 10). This final step of contrast enhancement resulted into a completely derivable image.

To implement this *Module 1* in the MATHEMATICA code, we used the following sequence of functions over the initial frame imagen:

GaussianFilter[ LocalAdaptativeBinarize[ ImageAdjust[ imagen ], n] n]

**References**

[https://reference.wolfram.com/language/ref/GaussianFilter.html](https://reference.wolfram.com/language/ref/GaussianFilter.html)

<https://reference.wolfram.com/language/ref/LocalAdaptiveBinarize.html>

<https://reference.wolfram.com/language/ref/ImageAdjust.html>

***Module 2:* Detection-description of the spots of interest, or key-points, by SURF.** To detect the chromatin spots and their relevant features during measurements, the previously processed stack of contrast-enhanced frames was introduced in the SURF module, which is schematically described below (further details in Supplementary Note 5):

- 1. *Region of interest:* We delimited a connected region of interest (ROI) inside the nucleus interior, excluding the nuclear membrane, visible nucleuoli or any other non-relevant intranuclear structure. This selection was made manually in the first frame of the sequence. The key-points were only detected in this ROI.
  2. *Key-points detection:* We detected the points of interest with the conditions of the biggest optical contrast, spherical shape and constant size conserved almost invariant along the sequence. SURF was implemented in MATHEMATICA with the function ImageKeypoints[ imagen, "PixelPosition", “Scale”, Method->"SURF", MaxPoints-> n , Masking->ROI], with *n* being the biggest number of key-points detected, typically *n* = 75. Using the algorithmics of the SURF scheme, the function ImageKeypoints[ ] found keypoints in the selected ROI of imagen and returned their features in a list (coordinates, size, orientation, contrast, etc..).
  3. *Key-point correspondence:* A matrix with the positions of the key-points detected was compared with the corresponding matrix obtained for the next sequence. A maximal tolerance of 5% was allowed to vary from this featuring matrix to the next one. Specifically, using a matrix of Euclidean distances, we determined which detected key points in a frame had correspondents in the next one within a tolerance in distance of 2 pixels. All points without a correspondent were discarded. We performed this analysis sequentially as a diagnosis of key-point correspondence between consecutive frames.
  4. *Gaussian profiling:* All potentially traceable key-points were analyzed as intensity spots in terms of shape invariance. The intensity profile of the detected key-points was fitted to 2D-Gaussians with just only one parameter of variance, i.e. representing spots with a circular symmetry. We rejected any spot with a tolerance higher than 5%, without a convergence after 100 steps of fitting, with spurious Gaussian widths either lower than 0.3 microns (below optical resolution) or larger than 1.5 microns (corresponding to big structures). To perform this analysis in MATHEMATICA, we used the function NonlinearModelFit[ ].

This *Module 2* identified chromatin spots with invariant features maintained within the prescribed tolerances: maximal constrast (positive or negative), sphericity, size and undergoing short steps between sequences. Any other spot initially detected is discarded for further tracking.

**References**

<https://reference.wolfram.com/language/ref/ImageKeypoints.htmlaling>

<https://reference.wolfram.com/language/ref/NonlinearModelFit.html>

***Module 3:* MPT of relevant spots (only circular-Gaussian profiles are allowed).** Once the relevant spots were detected (and their features described), we performed the analysis of MPT in compliance with the global correspondence of all the spots along the whole sequence of tracking. We used the MATHEMATICA function ImageFeatureTrack[ ], which exploits the biggest spatial correlations with Euclidean distances (using ImageCorrelate[ ]) to track the featured spots in the sequence of frames. The function ImageFeatureTrack[ ], automatically fixed the set of key-points to be tracked in the different frames and then returns a list with their positions along the sequence. The function ImageFeatureTrack [[MaxFeatureDisplacement](https://reference.wolfram.com/language/ref/MaxFeatureDisplacement.html), MaxIterations, Tolerance ] worked as a global MPT optimization schema under three fitting parameters: *i)* [MaxFeatureDisplacement](https://reference.wolfram.com/language/ref/MaxFeatureDisplacement.html) is the biggest displacement allowed for a point between consecutive frames. Similarly to the SURF module, we established a maximal displacement of 2 pixels, whicht avoided tracking active spots that undergo large displacements as Levy flights (if there exist here). *ii)* MaxIterations was the biggest number of iterations, fixed at 50 by defect although normally the function converges after 3-4 iterations. *iii)* Tolerance established as a number from 0 to 1 for global variances in the correspondence procedure. We fixed at Tolerance = 0.05, which obligated a maximum of 5% variance in the correlations between all the key-points in two consecutive frames. Any ImageFeatureTrack without the correspondent point in the next frame was discarded, and the trajectory was not analyzed. The outcome of ImageFeatureTrack was a matrix with the set of bare trajectories as a function of time for validated spots. After SURF detection (with a circular Gaussian profile and invariant features) as input for ImageFeatureTrack, practically all points were accepted for tracking along the whole sequence and the optimization procedure was finished by this *Module 3* after no more than 5 iterations.

**References**

<https://reference.wolfram.com/language/ref/ImageFeatureTrack.html>

<https://reference.wolfram.com/language/ref/ImageCorrelate.html>

***Module 4:* Drift correction.**This last module performed the final refinement of tracked trajectories by correcting the possible drifts due to global translational and rotational displacements of the nucleus as a whole. We used the MATHEMATICA modulus FindGeometricTransform, which defined the optimal geometric transformation to minimize the distances between two consecutive sets of key-points. For a given frame recorded at a time$t$, the function FindGeometricTransform[“Rigid”] applied the following rigid transformation on the position matrix $X$:

$X^{'}\left( t \right)=T+R_{\theta}X\left( t \right)$,

where $T$ is the shift operator accounting for a translation, and $R$ is the operator of rotation for the rotation angle $\theta$.

Then, the distance between two consecutive sets of coordinates $X^{'}\left( t \right)-X^{'}\left( t-1 \right)$ is minimized, this is:

$\delta\left| X^{'}\left( t \right)-X\left( t-1 \right) \right|=0$,

which allows for obtaining the parameters that define the best alignment between consecutive frames, i.e. the rigid transformation that better conserves the distances among the traced spots. The function FindGeometricTransform[“Rigid”, X(t-1), X(t) = X'(t) ] uses a translation-rotation transformation with a linear solver based on SVD (Singular Values Decomposition). We assumed that the whole nucleus behaves as a 2D-rigid body within this transformation, thus being globally represented by the coordinates of the center of mass (and two moments of inertia). The positions of the center of mass were considered as the drifting trajectory to be subtracted to the coordinates of any trajectory in the MPT outcome (Supplementary Figure S1b and Figure S2).

**Reference**

<https://reference.wolfram.com/language/ref/FindGeometricTransform.html>

**Supplementary Note 4. Experimental results with Jurkat nuclei.** All performance tests were based on the original implementations encoded in MATHEMATICA by the authors. The test of performance is separated in two sections refered to the SURF-only and MPT-SURF modules, respectively.

- 1. ***Image processing for detection-description chromatin spots (SURF performance).*** Supplementary Figure S2a (upper panels) shows a raw image of a Jurkat nuclei (Panel 1) that was pre-processed for further detection of the interest key spots (Panel 2). Local mobilities obtained from of chromatin spots provided the distribution of apparent microviscosities (Supplementary Figure S2b, see caption for details). The spots of interest detected in Panel 2 were splitted in Panel 3 as two families: dark and bright spots in left and right panel, respectively; and their viscosities calculated (Supplementary Figure S2b). The average values and standard deviations of the apparent viscosities were practically identical for bright and dark spots, or both.


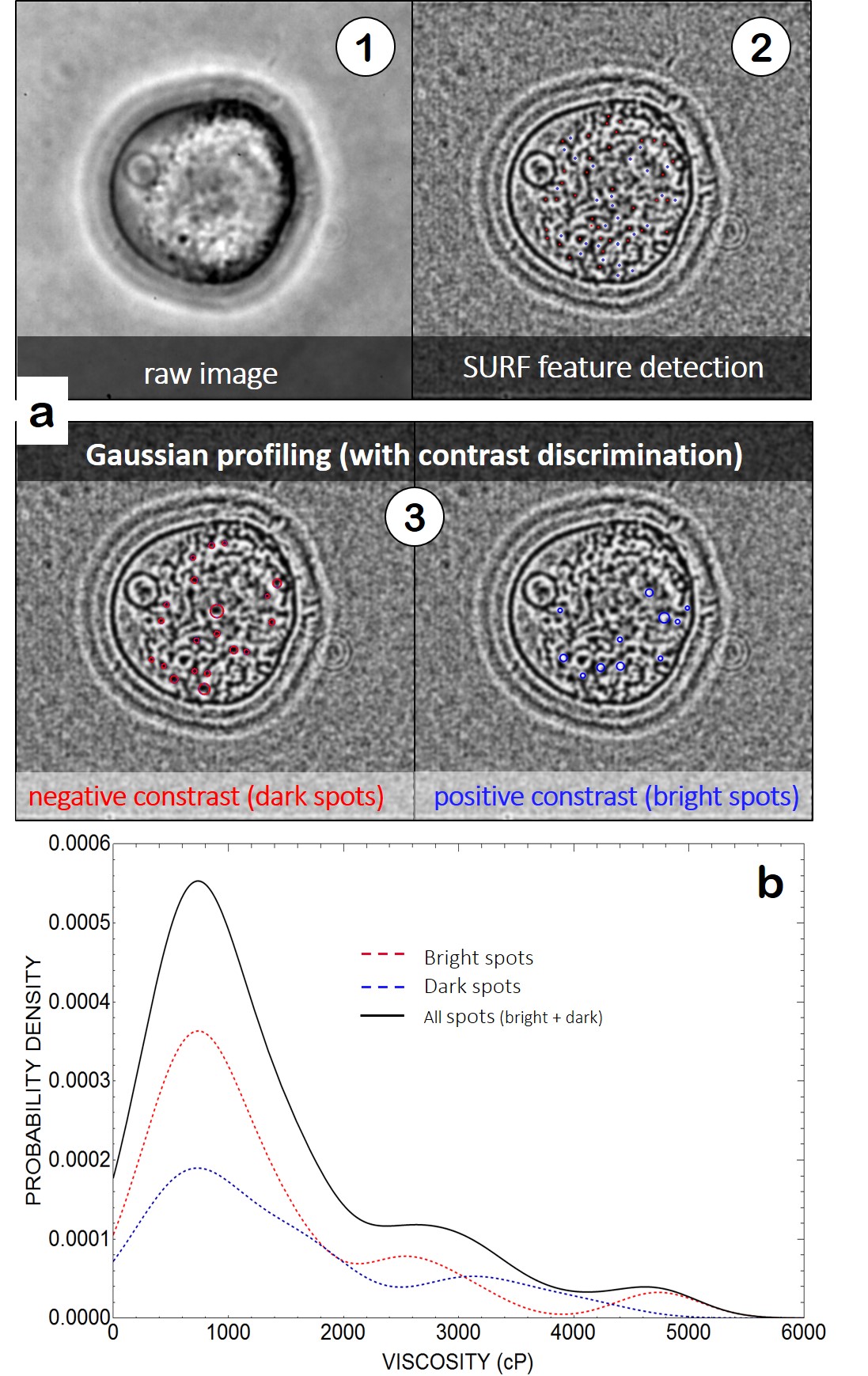


**Figure S2. (a)** SURF detection-description of chromatin spots from isolated Jurkat nuclei (1: Raw microscopy image; 2: SURF detection of spots of interest after contrast enhancement; 3: Spot description after Gaussian profiling). **(b)** Probability distribution of the apparent viscosities from chromatin spots detected in Figure S2a by MPT-SURF. Similar equivalence was observed for all the Jurkat nuclei studied ($N=17$).

- 1. ***Multiple particle tracking of SURF-featured chromatin spots (MPT-SURF performance).*** Supplementary Figure S3 shows how Module 3 of feature tracking based on the ImageFeatureTracking function (upper panels) coupled with the Module 4 of drift correction based on FindGeometricTransform (lower panels). Spot trajectories from Supplementary Figure S2 were represented in the central upper panel. The nuclear movement was observed as displacements (translations and rotations), as the weigthed average of all the trajectories (the optical intensity $I_{i}$ being the weighting factor). The instantaneous position of the center of mass was determined with coordinates:

$x_{CM}\left( t \right)=\frac{\sum_{i} I_{i}\left( t \right)\boldsymbol{x}_{\boldsymbol{i}}\left( t \right)}{\sum_{i} I_{i}\left( t \right)}$,

where *i*-index referred to the spots of interest detected. Larger and deterministic drifts might be processed efficiently by the method of geometric transformation. Then, spot trajectories were subjected to drift-correction by subtraction of the averaged motion of all the trajectories. Supplementary Figure S3 shows an unique profile for all the frames along the sequence (central lower panel), a single point for the corrected center-of-mass (left lower panel) and a set of spot trajectories more compatible with the confined diffusion expected in the highly crowded chromatin environment (right lower panel). These results validated the set of SURF-featured spots detected in the chromatin as a frame-of-reference adequate for effective drift correction.


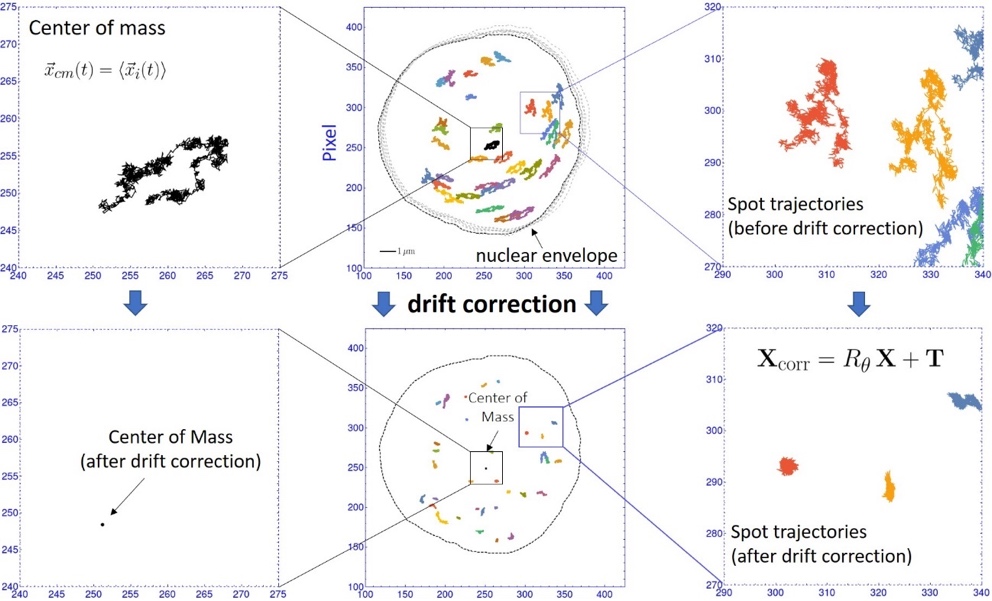


**Figure S3.** MPT-SURF analysis of the spots of interest detected in a representative Jurkat nucleus: Before drift-correction (upper panels); After drift-correction (lower panels).

- 1. ***Feature invariace with drift-corrected MPT-SURF.*** The size and the optical density (measured by the average intensity) of each spot had to be constant for the tracking schema. Spot sphericity was assured as a precondition of SURF algorithm, spots with non-circular intensity profiles were discarded. Supplementary Figure S4 shows time-traces for instantaneous size and intensity of three spots, which undergo long (panel 1 and 2) and small (lower panel 3) displacements. In all cases, both variables fluctuated below the 5% tolerance prescribed by MPT-SURF. The size change remained below a fraction of the pixel size. No drift was observed in spot features during experiments, which was required for the microrheological analysis.


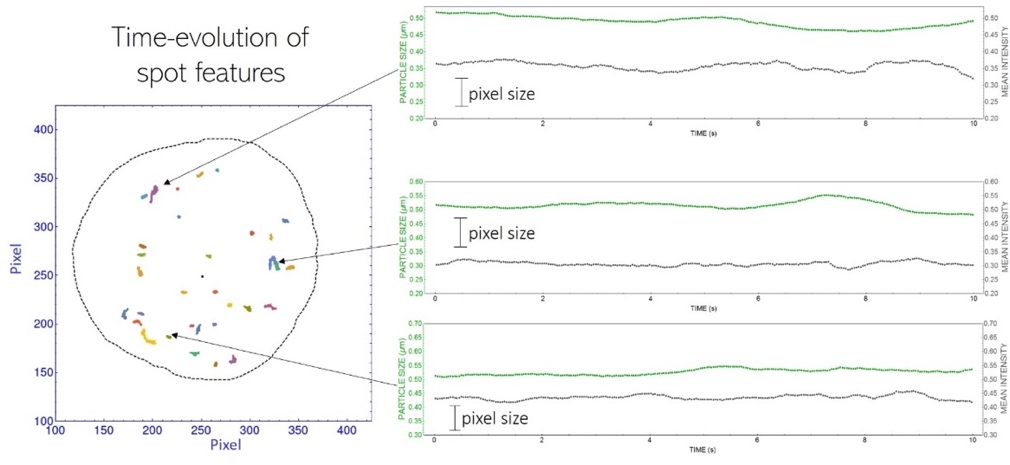


**Figure S4. Feature invariance with MPT-SURF.** Drift correction of the size (green line) and the optical density (grey line) of the spots detected by MPT-SURF. These features vary by less than the general 5% processing tolerance imposed to the MPT-SURF procedure.

Supplementary Figure S5 shows the spot size variability relative to the initial value for each time. To gain a statistical significance, values were averaged for all nuclei studied (including all the cell types). No significant drift was observed in spot size during 1min-measurements. Statistical-distributed fluctuations remained below 15%. These were due to the biological variability between different specimens, as the error within a single nucleus was determined by MPT-SURF method (5%-tolerance).


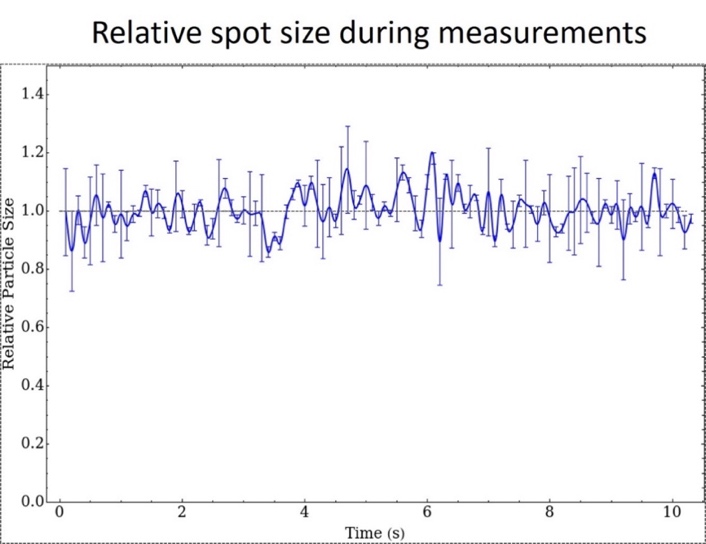


**Figure S5.** Variance band of the relative spot sizes during tracking experiments using the MPT-SURF algorithm. The relative size was defined with respect to the initial value determined for a given spot. The average values were calculated for a collection of more than 150 nuclei obtained from the different cell types. Each nucleus was characterized by an average size spot obtained from more than 30-40 featured chromatin spots. The standard deviations in the plot were calculated over more than 5000 data per time-lapse.

***4.4) Compared analysis with Jurkat cells: Isolated ex-cell vs. intact in-situ nucleus.*** To test the MPT-SURF method with the analytic platform proposed, we compared the apparent viscosities measured from isolated nuclei and those nuclei in intact Jurkat cells. Supplementary Figure S6a (upper panels) shows that chromatin spots in isolated nuclei were more crealy identifiable than in intact cells. However, MPT-SURF algorithm detected a similar number of featured spots susceptible to be tracked in both cases (Supplementary Figure S6a, lower panels). The Brownian dynamics of those chromatin spots depicts similar qualitative features in both cases: i) effective confinement characterized by asymptotic free-diffusivity at very short lag-times (Supplementary Figure S6b) and ii) active hyperdiffusivity at long lag-times (Supplementary Figure S6c). Using MSD-plots like those shown in Supplementary Figure S6b, we obtained the effective values of the diffusion coefficient $D_{eff}$ from the best fits to the linear function in Eq. (1); then, the distributions of apparent viscosities were calculated using Eq. (2). By taking adavantage of this calculation schema, similar viscosity distributions were found in isolated nuclei or *in situ* nuclei of Jurkat cells (Supplementary Figure S6d). The differences observed in each case were not statistically significant; however, we estimated a mean value slightly higher for isolated nuclei ($\eta_{app}^{\left( in \right)}=280\pm200 cPoise, N=17$) than for intact cells ($\eta_{app}^{\left( ex \right)}=210\pm150 cPoise, N=24$).


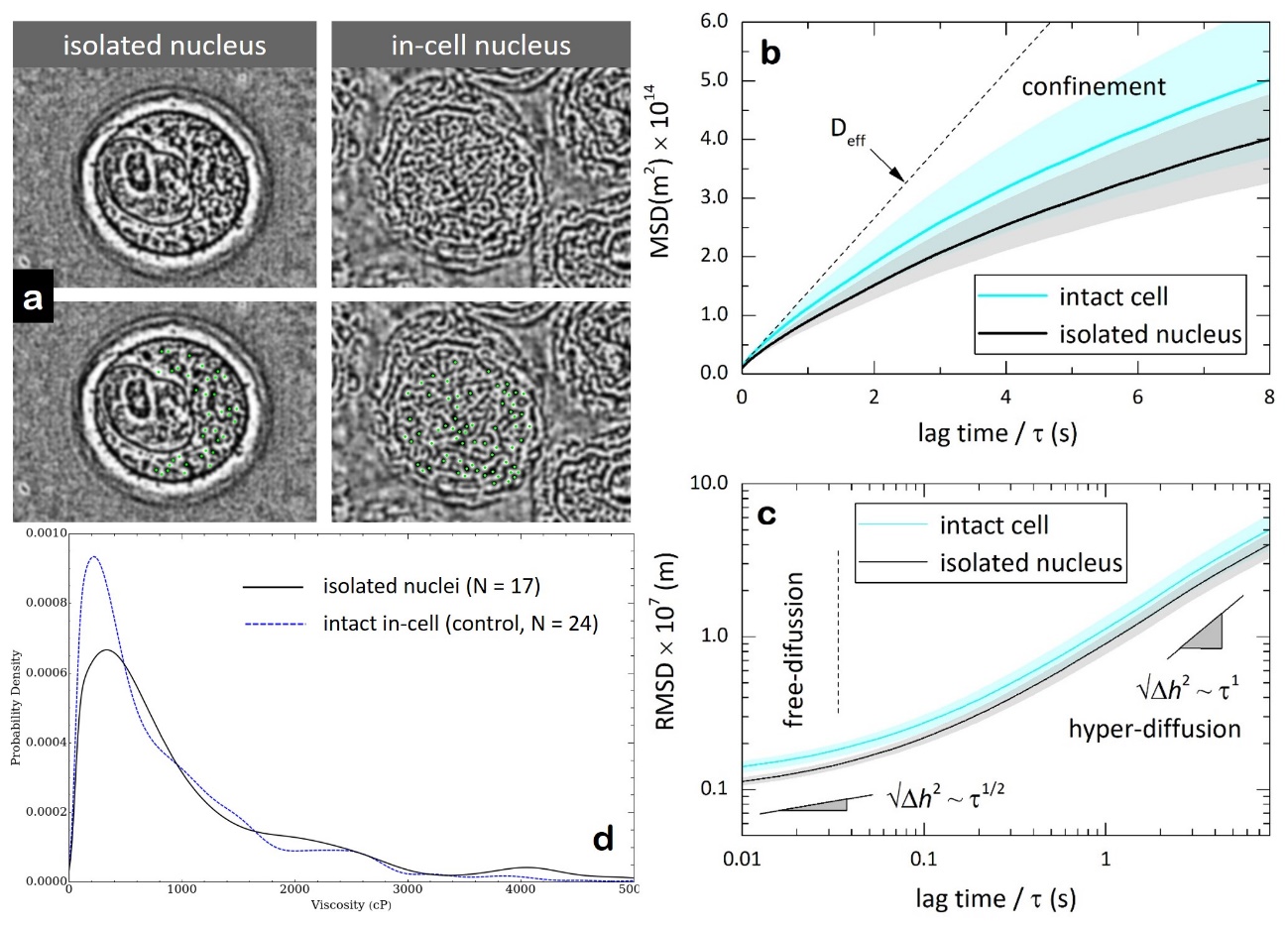


**Figure S6. (a)** Comparison of MPT-SURF-based analysis of nuclei upon isolation or in intact cells. **(b)** Linear MSD-plot of the Brownian mobilities detected in-cell for chromatin spots in an intact nucleus of a Jurkat cell (blue symbols). Comparison with ex-cell measurements in an isolated nucleus (black/gray symbols). The straight lines correspond to averaged values and the shadow regions to the variability band within the chromatin spots tracked in every nucleus. **(c)** Linearized log-log plot of the RMSD’s shown in panel b). **(d)** Statistical distribution of the apparent viscosities measured in both cases for respective nuclei populations ($N=17$ for isolated nuclei; $N=24$ for intact nuclei).


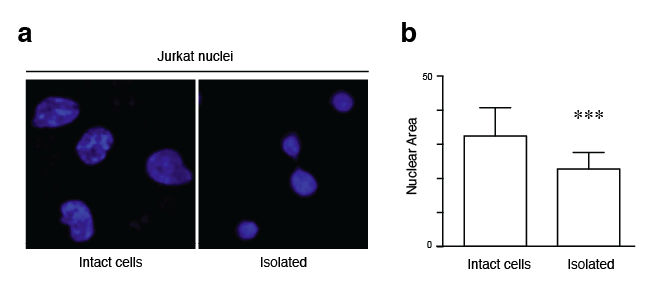


**Figure S7. (a)** Nuclei after isolation or in intact cells were stained by Hoechst 33342. **(b)** Nuclear area was determined by confocal microscopy. N>35 nuclei. *** P <0.001.

~~
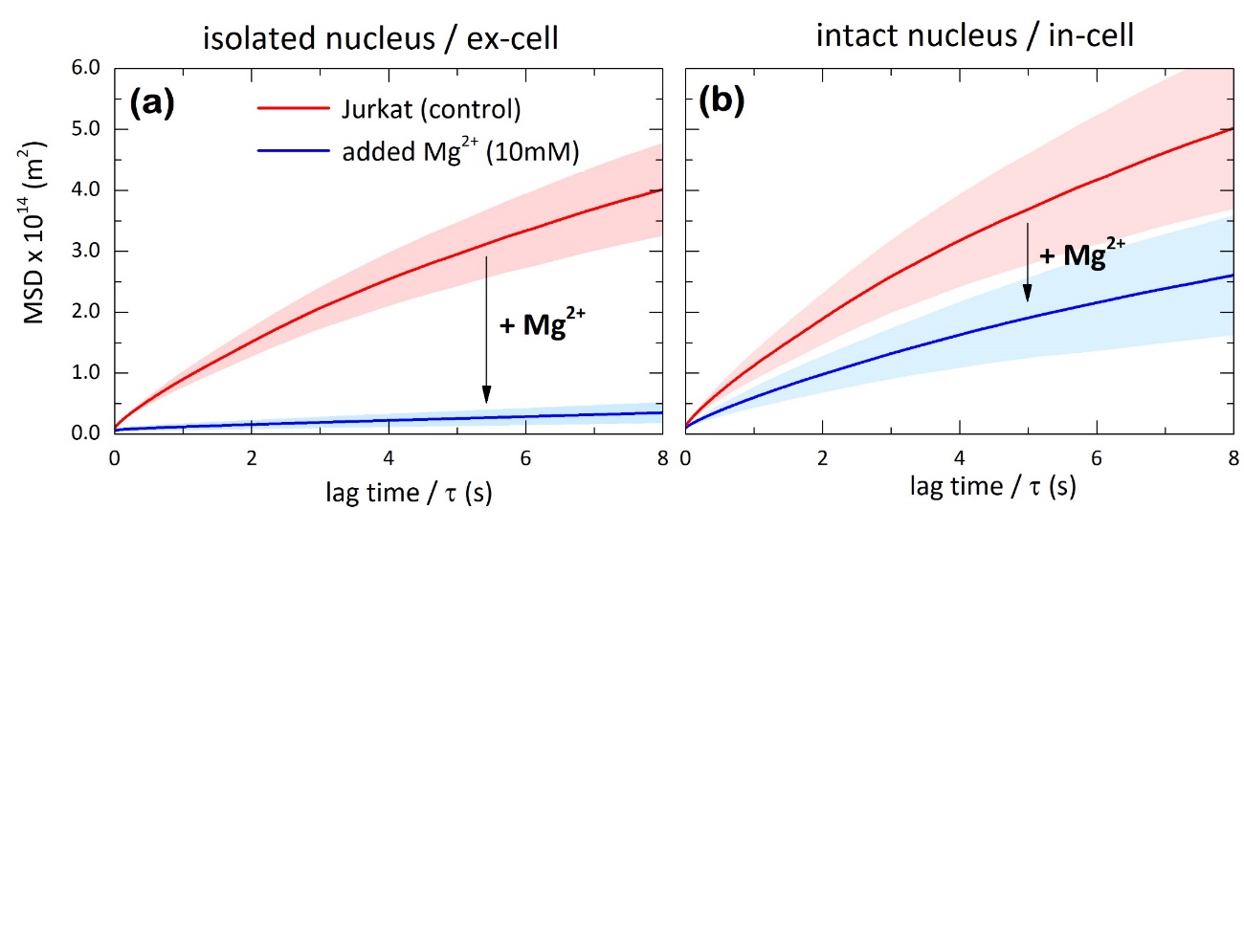
~~

**Figure S8.** Effect of Mg^2+^ addition on the diffusion trajectories of the chromatin granules in isolated nuclei **(a)** or intact Jurkat cells **(b)**. The dashed regions represent the variability band of the chromatin mobilities within a single nucleus; averaged mobility represented by straight lines (red symbols: untreated condition; blue symbols: added Mg^2+^).


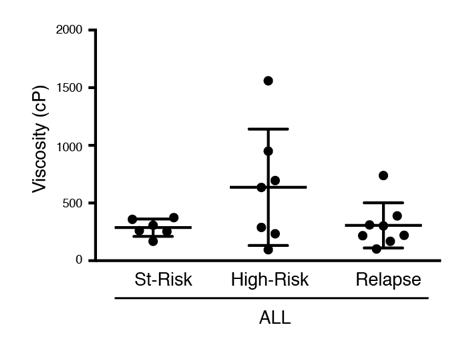


**Figure S9.** Intact cells from Standard- (St); High-(High) risk; and relapsed ALL patients were analyzed by MPT-SURF technology and the apparent viscosities determined by passive microrheology.

**Supplementary Note 5. Speeded-up Robust Features (SURF) detector-descriptor.** The SURF algorithm is a well knwon detector/descriptor of scale- and rotation-invariant particles in digital images [13]. The tolerance of the analysis was defined by constant geometry and photometry of the images. The nucleus might present translational and rotational geometric changes that induced drifts in the trajectories of the single granules detected. Photometric changes by internal fluctuations of the detected granules might affect the particle tracking. Using our SURF implementation, we focused on scale and image rotation invariant detectors and descriptors that confirmed complexity and robustness to these commonly occurring deformations. This performance was achieved by relying on integral images for image convolution that applied a Hessian matrix-based measure for the detector [^[[15]](#endnote-15)^] and a SIFT analysis, which describes spatial intensity patterns [^[[16]](#endnote-16)^].

**1)** **SURF Detector:** The SURF detector was based on the Hessian matrix $H\left( \boldsymbol{x},\sigma\right)$ defined in terms of the coordinates $\boldsymbol{x=}\left( x,y \right)$ and the scale $\sigma$, which fixes the spatial resolution of the filter used to determine the local correspondence of the points of interest. Particularly, for a given image the Hessian matrix is defined as:

$H\left( x,\sigma\right)=\left[ \begin{matrix} \partial_{xx}^{2} & \partial_{xy}^{2} \\ \partial_{yx}^{2} & \partial_{yy}^{2} \end{matrix} \right]$,

which determines the local curvature of every point of interest with coordinates $\boldsymbol{x}$ placed at the center of a pixel grid of scale $\sigma$. The trace of the Hessian matrix represents the mean curvature of the image around the point of interest (Laplacian, or extrinsic curvature), and the determinant is the Gaussian curvature (intrinsic curvature). As a filter of the intensity around each point of interest (a chromatin spot), SURF uses the Gaussian profile that best fits an embedding square-box grid of $9\times9$ pixels (the 80 pixels that exist around a given point of interest placed at $\boldsymbol{x}$). The Gaussian profile is chosen to reasonably approach the optical aspect of a spherical particle and to have a well-defined discretized representation that allows for easy digitalization of the second order derivatives (see Fig. 1 in ref. [13]). The $9\times9$-box filters allow for embedding Gaussian second-order derivatives with $\sigma=1.2$, which represents the lowest scale in a correspondence scheme that searchs for spot feature congruence at larger scales. In order to analyse local correspondences with SURF, higher scales are rescaled-up as pyramids (e.g. a higher-scale $27\times27$ filter is actually a $3\times3$-box made of minimal $9\times9$ (pixel)-boxes; in this case, the scale parameter is $\sigma=3\times1.2=3.6$, and so on). The integral image around a given point of interest is analyzed by up-scaling the filter size rather than by zooming the image size (only 64 dimensions are used up to maximal grid of $192\times192$ pixels). In SURF, the “Fast-Hessian” detector localized spots of interest by performing a Gaussian fit in each $9\times9$-box around every pixel in the image ($\sigma=1.2$). Then, SURF determined the maximum of the determinant of the Hessian matrix, which identified the points with a maximum Gaussian curvature (maximal optical contrast around) in this lowest scale ($\sigma=1.2$). The detection process was then rescaled-up ($\sigma=3.6, \ldots$), which identified the scale $s=\sigma_{inv}^{\left( tol \right)}$ to keep constant Gaussian features (at 5% of tolerance for the averaged value). We identified the center and the width of the Gaussian filter as the features to remain scale-invariant for every point of interest. Once the group of potential points of interest were identified, the biggest differences of the Hessian matrix were then interpolated in space- and image-space using the method proposed by Brown [^[[17]](#endnote-17)^]. This group of “percolated” points of interest in a given image was considered as the feature to be conserved in the correspondence analysis with the subsequent frame in the lapse-time analysis of the trajectories.

**2)** **SURF Descriptor:** We defined the ROI of each point as an specific and constant (to small deformations, localisation errors and different images) vector. This descriptor was defined from a square region of the reproducible orientation obtained from a circular ROI. The SIFT algorithm [^[[18]](#endnote-18)^] identifies a reproducible orientation for the point of interest based on the Haar-wavelet responses calculated in two orthogonal directions [^[[19]](#endnote-19)^]. The SIFT-descriptor of SURF computed a histogram of local oriented gradients of ROIs (radius $6s$ around the point of interest, with $s$ being the scale of the point of interest) and stored the bins in a 128-dimensional vector (8 orientation bins for each of the $4\times4$location bins). Once the wavelet responses were calculated and weighted with a Gaussian ($\sigma=2.5s$) centered at the point of interest, responses were represented in a vectorial space with the horizontal response strength of the wavelet along the abscissa and the vertical response strength along the ordinate. The good performance of SIFT compared to other descriptors is remarkable [18]; however, its high dimensionality is a drawback at the final matching step. The goal of spots description was to obtain numerical representations of the location and properties of their image features. Image features were the local biggest or smallest intensities significantly different from their neighborhood. Following the standard defined by Jaqaman and Danuser [14], we compared foreground-to-background intensity as a statistical test in which the intensity presented a value higher than three times the background ($SNR\geq3$ ). We further described the spots by the sign of their optical contrast, i.e. by the sign of the Laplacian as measured by the trace of the Hessian matrix. Typically, the interest spots were found as granule-type structures, either as bright spots on dark backgrounds (probably corresponding to dense heterochromatin) or dark spots on bright backgrounds (probably corresponding to euchromatin regions). This feature was available at no extra computational cost, as it was already computed during the detection phase.

**3)** **MPT Matching:** Once the chromatin spots by frame were detected/described by SURF, the MPT defined particle trajectories throughout the time-lapse sequence of frames. Multiple factors, as high particle density, spot heterogeneity, temporary disappearance (e.g. resulting from out-of-focus motion and detection failure), particle merging and trajectory exchange (i.e. two particles approaching each other), and particle splitting (i.e., two unresolved particles diverging to resolvable distances) hinder the correspondence between frames [14]. To avoid these problems only spots with near-invariant features were considered (within the ±5% of admited variance). To match the correspondence between consecutive frames, we determined the vectorial map of orientations of the $6s$- neighborhood around each spot of interest (see above). Spots with a change of neighborhood orientation higher than 5% were rejected for further MPT analysis (at least 95% overlapping required). Although the two types of contrasted spots (bright and dark) were indistinctly tracked, we only considered those with the same type of contrast (no trajectory crossover is allowed).

1. [] Mason, T., Ganesan, K., van Zanten, J.H., Wirtz, D. and Kuo, S.C. Particle tracking microrheology of complex fluids. Phys. Rev. Lett. 79(17), 3282-3285 (1997). [↑](#endnote-ref-1)
2. [] Yamada, S., Wirtz, D., and Kuo, S.C. Mechanics of living cells measured by laser tracking microrheology. Biophys. J. 78(4), 1736-1747 (2000). [↑](#endnote-ref-2)
3. [] Luby-Phelps K. Taylor D.L. Lanni F. Probing the structure of cytoplasm. J. Cell Biol. 102: 2015-2022 (1986). [↑](#endnote-ref-3)
4. [] Hou L., Luby-Phelps K. and Lanni F. Brownian motion of inert tracer macromolecules in polymerized and spontaneously bundled mixtures of actin and filamin. J. Cell Biol. 110, 1645-1654 (1990). [↑](#endnote-ref-4)
5. [] Xu, J., A. Palmer, and D. Wirtz. Rheology and microrheology of semiflexible polymer solutions: actin filament networks. Macromolecules. 31:6486–6492 (1998) [↑](#endnote-ref-5)
6. [] Mason, T. G., A. Dhople, and D. Wirtz. Concentrated DNA rheology and microrheology. Mat. Res. Soc. Symp. Proc. 463:153–158 (1997). [↑](#endnote-ref-6)
7. [] <http://web.mit.edu/savin/Public/.Tutorial_v1.2/Introduction.html> [↑](#endnote-ref-7)
8. [] Crocker, J.C. and Hoffman, B.D. Multiple‐Particle Tracking and Two‐Point Microrheology in Cells. Meth Cell Biol. 83, 141–178 (2008). [↑](#endnote-ref-8)
9. [] Meijering, E., Dzyubachyk, O. and Smal, I. Methods for Cell and Particle Tracking. In Imaging and Spectroscopic Analysis of Living Cells. Methods in Enzymology, vol 504, pp. 183-200, edited by P. M. Conn, Elsevier (2012) [↑](#endnote-ref-9)
10. [] Manzo, C. and García-Parajo, M.F. A review of progress in single particle tracking: from methods to biophysical insights. Rep. Prog. Phys. 78(12), 124601 (2015) [↑](#endnote-ref-10)
11. [] Swedlow JR, Goldberg I, Brauner E, Sorger PK. Informatics and Quantitative Analysis in Biological Imaging. Science 300(5616), 100–102 (2003) [↑](#endnote-ref-11)
12. [] Eils R, Athale C. Computational imaging in cell biology. J Cell Biol. 161(3), 477–481 (2003). [↑](#endnote-ref-12)
13. [] Bay, H., Ess, A., Tuytelaars, T. & Van Gool, L. SURF: Speeded-up robust features. Comput Vis Image Underst 10, 346-359 (2008). doi: 10.1016/j.cviu.2007.09.014 [↑](#endnote-ref-13)
14. [] Jaqaman, K. and Danuser, G. Computational image analysis of cellular dynamics: A case study based on particle tracking. Cold Spring Harb. Prot. 4(12), 1-10 (2009). [↑](#endnote-ref-14)
15. [] Mikolajczyk, K., Schmid, C.: Indexing based on scale invariant interest points. Internat. Conf. Comput. Vision 1, 525-531 (2001): <https://hal.inria.fr/inria-00548276/document> [↑](#endnote-ref-15)
16. [] Lowe, D.: Distinctive image features from scale-invariant keypoints, cascade filtering approach. Int. . Compt. Vision 60, 91-110 (2004): [↑](#endnote-ref-16)
17. [] Brown, M. and Lowe, D. Invariant features from interest point groups. In: BMVC (2002): <https://www.cs.ubc.ca/~lowe/papers/brown02.pdf> [↑](#endnote-ref-17)
18. [] Mikolajczyk, K., Schmid, C.: A performance evaluation of local descriptors. IEEE Trans. Pattern Anal. Machine Intelig. 27(10), 1615–1630 (2005) [↑](#endnote-ref-18)
19. [] Chui, C.K. An Introduction to Wavelets (Academic Press, San Diego, 1992). ISBN 0-585-47090-17 [↑](#endnote-ref-19)
